# Common hematological and biochemical parameters for predicting urinary tract infections in geriatric patients with hip fractures

**DOI:** 10.3389/fmed.2024.1333472

**Published:** 2024-05-30

**Authors:** Wanyun Tang, Wei Yao, Wei Wang, Qiaomei Lv, Wenbo Ding, RenJian He

**Affiliations:** ^1^Department of Orthopedics, Zigong First People’s Hospital, Zigong, China; ^2^Department of Orthopedics, Dandong Central Hospital, China Medical University, Dandong, China; ^3^Department of Oncology, Dandong Central Hospital, China Medical University, Dandong, China

**Keywords:** hip fracture, urinary tract infection, geriatrics, hematological and biochemical parameters, predicting

## Abstract

**Background:**

This study aims to discern the significance of common hematological and biochemical parameters for predicting urinary tract infections in geriatric patients with hip fractures.

**Methods:**

Multivariable logistic regression and propensity score-matched analyses were conducted to calculate adjusted odds ratios (ORs) and 95% confidence intervals (CIs) for UTIs. The abilities of these parameters to predict UTIs were evaluated by receiver operating characteristic (ROC) curves. Dose–response relationships were assessed by categorizing hematological and biochemical parameters into quartiles. Subgroup analyses were further explored to investigate the relationship between these parameters and urinary tract infections.

**Results:**

Out of the 1,231 participants, 23.2% were diagnosed with UTIs. Hyperglycemia, hypoproteinemia and hyperglobulinemia were risk factors for UTIs in multivariate analysis. After propensity score matching, hyperglycemia (OR 2.14, 95% CI 1.50–3.05, *p* < 0.001), hypoproteinemia (OR 1.75, 95% CI 1.18–2.63, *p* = 0.006), and hyperglobulinemia (OR 1.38, 95% CI 0.97–1.97, *p* = 0.074) remained significantly associated with increased odds of urinary tract infections. ROC curve analyses showed moderate predictive accuracy of blood glucose, albumin and globulin for UTIs, with areas under the curves of 0.714, 0.633, and 0.596, respectively. Significant dose–response relationships were observed between these parameters and UTIs. The associations were consistent in subgroup analyses.

**Conclusion:**

Blood glucose, albumin and globulin levels can facilitate early identification of geriatric hip fracture patients at high risk of UTIs. These easily obtainable hematological and biochemical parameters provide a practical clinical prediction tool for individualized UTI prevention in this population.

## Introduction

Urinary tract infection (UTI) is a common but often underestimated complication among geriatric patients with hip fractures (1, 2). The reported incidence of UTI ranges from 8 to 52% during hospitalization for hip fractures (1–4). UTI increases the risks of delirium, mortality, and other postoperative complications in these patients (5, 6). It also hampers rehabilitation progress and functional recovery (7). Additionally, UTI significantly prolongs hospital stays and increases medical costs in elderly hip fracture patients (8).

In recent years, several studies have suggested that hematological and biochemical parameters obtained from routine blood tests may help predict UTIs (9–11). Hematological and biochemical parameters, such as blood glucose, white blood cell count, and hemoglobin levels, reflect the inflammatory state and immune function of the body, which may be associated with the occurrence and severity of UTIs (12). Beloosesky et al. found that elevated high-sensitivity C-reactive protein (hs-CRP) levels and increased white blood cell count were associated with a higher incidence of UTIs in geriatric hip fracture patients (13). Yao et al. demonstrated a positive correlation between elevated blood glucose levels and increased UTI risk (3). Despite some preliminary findings suggesting the potential value of hematological and biochemical parameters in predicting urinary tract infections in geriatric hip fracture patients, the current research exploring the relationship between different hematological and biochemical parameters and UTIs in this population is still limited and inconsistent.

This study aims to investigate the association between the common hematological and biochemical parameters with UTI occurrence in geriatric patients with hip fractures through a retrospective analysis of a large patient cohort. These hematological and biochemical parameters provide clinicians with a simple, reliable tool to identify patients at higher UTI risk early. This research will contribute to improved patient outcomes, reduced healthcare costs, and enhanced quality of care for geriatric patients with hip fractures.

## Methods

### Patient selection

A retrospective collection of clinical data was performed for 2,387 patients who had hip fractures at the Affiliated Dandong Central Hospital of China Medical University between July 01, 2011 and July 01, 2023. This study followed the STROCS guideline and respected the principles of the 1964 Helsinki Declaration and its subsequent amendments. The Institutional Review Board of Dandong Central Hospital approved the study. The studies were conducted by the local legislation and institutional requirements. Written informed consent for participation was not required from the participants or the participants’ legal guardians/next of kin in accordance with the national legislation and institutional requirements.

Inclusion Criteria: (1) age ≥ 60 years; (2) radiographic confirmation of hip fracture through X-ray or CT imaging; (3) surgical confirmation of hip fracture. Exclusion Criteria: (1) Age < 60 years old, (2) Patients with multiple, old and pathological hip fractures, (3) Patients who did not receive surgical treatment, (4) Patients with a history of recent (within the past 3 months) antibiotic use, urinary catheterization, or recurrent UTI, (5) Incomplete or missing hematology date for any reason. After applying the exclusion criteria, 1,156 patients were excluded, leaving a final retrospective cohort of 1,231 patients for analysis. A flow diagram depicting the screening process and participant selection is provided in Supplementary Figure 1.

### Diagnostic criteria of UTI and outcome

The primary endpoint was the incidence of UTIs during hospitalization. UTI diagnoses were established per Centers for Disease Control (14) and Prevention criteria, necessitating at least one clinical sign (fever over 38°C, dysuria, increased voiding frequency/urgency, or suprapubic tenderness) coupled with evidence of infection from positive urine culture (over 105 CFU/mL) or urine analysis (revealing leukocyte esterase and nitrites). Two experienced urologists comprehensively evaluated the clinical and laboratory information to make definitive UTI diagnoses.

### Variables and data collection

We identified 45 hematological and biochemical parameters by reviewing relevant literature on previously studied hematological variables related to urinary tract infections (UTIs), and cross-referencing with the routinely assessed hematological tests at our institution (2, 9, 15–17). Demographic factors included age, gender, smoking, and alcohol use. Comorbidities considered were hypertension, diabetes, chronic liver disease, chronic kidney disease, prostate hyperplasia, urolithiasis, vesicoureteral disease, catheterization, total time of indwelling catheter, tumor. Injury variables encompassed fracture type, treatment type, bedridden time (from admission to surgery), surgery time, and the American Society of Anesthesiologists score (ASA). Hematological and biochemical parameters, obtained within 24 h of admission, included red blood cells, white blood cell, neutrophils, lymphocytes, platelets, hemoglobin, mean platelet volume, red cell distribution width, potassium, sodium, calcium, blood glucose, blood urea nitrogen, creatinine, uric acid, alanine aminotransferase, aspartate aminotransferase, total protein, albumin, globulin, cholesterol, low-density lipoprotein, high-density lipoprotein, triglycerides. Data retrieval from electronic medical records of orthopedic patients at Dandong Central Hospital was performed by three researchers (WYT, WY, QML), who were specifically trained in data collection.

### Statistical analysis

Continuous variables were presented as mean (standard deviation) and compared by Student’s *t*-test. Categorical variables were expressed as frequency and percentage (n, (%)) and analyzed by chi-square test or Fisher’s exact test. To eliminate the bias caused by missing data, we performed multiple imputation techniques for adjustment.

The multivariate logistic regression model included covariates with a *p*-value of <0.10 from the univariate analysis. The results of the model are presented as odds ratios (ORs), 95% confidence intervals (CIs), and corresponding *p*-values, with a significance level set at *p* < 0.05.

For variables displaying statistical significance (*p* value <0.05) within the hematological and biochemical parameters, we employed dichotomization and referenced the normal or recommended values found in pertinent literature to categorize the samples into two distinct groups. To delve deeper into our analysis, we utilized the nearest neighbor matching algorithm for propensity score matching analysis (17), implementing a caliper width of 0.25 standard deviations (SD) to ensure balanced matching. All covariates were meticulously matched in a 1:1 ratio between the groups. The assessment of differences in characteristics between the two groups was conducted using the standardized mean difference (SMD).

We constructed prediction models for hematological and biochemical parameters and urinary tract infection incidence and evaluated model performance by calculating the area under the curve (AUC). Additionally, we performed dose–response analyses of hematological and biochemical parameters to better characterize the associations between hematologic indices and risk of urinary tract infection. According to the quartile distribution of hematological indicators, patients were divided into four groups: Q1, Q2, Q3 and Q4. Sensitivity analysis assessed the robustness of the relationship.

Furthermore, we also conducted subgroup analyses to investigate the interactions between different levels of hematological and biochemical parameters and covariates in the model. The subgroup analysis results revealed the differences of hematological and biochemical parameters and urinary tract infection relationship in different populations, providing more comprehensive research conclusions.

All statistical analyses were performed using SPSS version 26 (developed by SPSS Inc) and R software version 4.0.3 (using Matching and Frailty packages developed by R Foundation for Statistical Computing).

## Results

**Figure 1:**
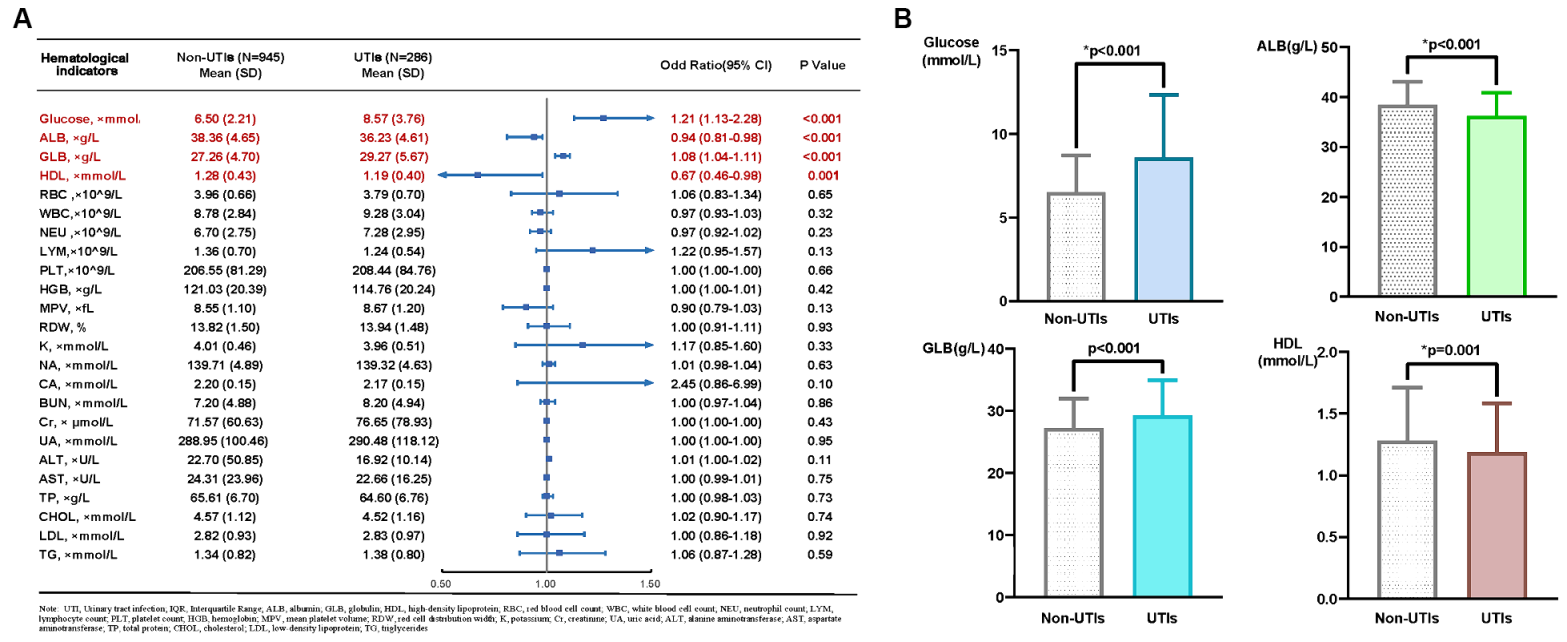
**(A)** Mean and *p*-value of hematological and biochemical indicators in patients with non UTIs and UTIs, **(B)** Disparities between four hematological and biochemical parameters in patients with non UTIs and UTIs.

Among the 1,231 patients in our retrospective study, 286 were diagnosed with UTIs. Table 1 shows the baseline demographic characteristics of patients with and without urinary tract infections. The collected Common hematological and biochemical parameters, along with their respective baseline levels, are depicted in Figure 1A. The glucose (*p* < 0.001), ALB (*p* < 0.001), GLB (*p* < 0.001) and HDL (*p* = 0.001) values were all significantly elevated in the UTIs group, as illustrated in Figure 1B.

**Table 1:** Baseline demographic characteristics of patients with and without urinary tract infections (UTIs).

	Total (*n* = 1,231)	Without UTIs (*n* = 945)	With UTIs (=286)	*p*-value
Demographic				
Age, × years (Mean, SD)	74.83 (9.60)	73.88 (9.59)	77.96 (8.94)	0.001
Female gender (n,%)	745 (60.5)	533 (56.4)	212 (74.1)	<0.001
Smoking (n,%)	209 (17.0)	176 (18.6)	33 (11.5)	0.005
Alcohol (n,%)	143 (11.6)	123 (13.0)	20 (7.0)	0.005
Comorbidities				
Hypertension (n,%)	625 (50.8)	441 (46.7)	184 (64.3)	<0.001
Diabetes (n,%)	289 (23.5)	174 (18.4)	115 (40.2)	<0.001
Chronic liver disease (n,%)	57 (4.6)	39 (4.1)	18 (6.3)	0.127
Chronic kidney disease (n,%)	64 (5.2)	33 (3.5)	31 (10.8)	<0.001
Prostate hyperplasia (n,%)	27 (2.2)	15 (1.6)	12 (4.2)	0.008
Urolithiasis (n,%)	18 (1.5)	10 (1.1)	8 (2.8)	0.032
Vesicoureteral disease (n,%)	57 (4.6)	27 (2.9)	30 (10.5)	<0.001
Catheterization (n,%)	570 (46.3)	393 (41.6)	177 (61.9)	<0.001
Total time of indwelling catheter ()	1.79 (3.46)	1.26 (2.25)	3.55 (5.54)	<0.001
Tumor (n,%)	112 (9.1)	89 (9.4)	23 (8.0)	0.478
Operation				
Fracture type				
Femoral neck fracture (n,%)	638 (51.8)	525 (55.6)	113 (39.5)	<0.001
Intertrochanteric fracture (n,%)	519 (42.2)	367 (38.8)	152 (53.1)	
Subtrochanteric fracture (n,%)	74 (6.0)	53 (5.6)	21 (7.3)	
Treatment type				
Total hip arthroplasty (n,%)	156 (12.7)	123 (13.0)	33 (11.5)	<0.001
Hemiarthroplasty (n,%)	297 (24.1)	231 (24.4)	66 (23.1)	
Intramedullary nail (n,%)	414 (33.6)	289 (30.6)	125 (43.7)	
Plate/screw (n,%)	167 (13.6)	120 (12.7)	47 (16.4)	
Multiple screws (n,%)	197 (16.0)	182 (19.3)	14 (5.2)	
Bedridden time, × days (Mean, SD)	5.88 (3.98)	5.57 (3.79)	6.90 (4.42)	0.026
Surgery time, ×hours (Mean, SD)	1.66 (0.80)	1.63 (0.77)	1.74 (0.88)	0.126
ASA				
III-V (n,%)	697 (56.6)	515 (54.5)	182 (63.6)	0.006
I-II (n,%)	534 (43.4)	430 (45.5)	104 (36.4)	

Data are presented as median (Mean, SD) and n (%).

ASA, American Society of Anesthesiologists score.

The Supplementary Table 1 shows the comparison of the incidence of UTIs before and after PSM based on different hematological indicators. There are similar baseline characteristics between the groups of the clinical cutoffs after PSM, indicating that matching has balanced the covariates well between the two groups (Supplementary Tables 3, 5, 7, 9). Compared to normal values, abnormal blood glucose (OR 3.88, *p* < 0.001), albumin (OR 2.00, *p* < 0.001), globulin (OR 1.65, *p* = 0.003), and HDL cholesterol (OR 1.47, *p* = 0.009) were associated with significantly increased odds of developing urinary tract infections. Details of the univariate and multivariate logistic regression analyses for UTIs are presented in Supplementary Tables 2, 4, 6, 8. Even after multiple regression adjustments, the results remain robust, showing that patients blood glucose (OR 1.90, *p* = 0.028), ALB (OR 0.68, *p* = 0.062), and GLB (OR 1.77, *p* = 0.023) are at increased risk of UTIs. After propensity score matching (PSM), the associations of glucose (OR 2.14, *p* < 0.001) and albumin (OR 0.68, *p* = 0.062) with urinary tract infections (UTIs) remained robust. However, the relationships of HDL (OR 0.84, *p* = 0.322) and globulin (OR 1.38, *p* = 0.074) with UTIs were attenuated after PSM (Table 2).

**Table 2:** Unadjusted and adjusted associations between UTIs and hematological indicators based on different cut-off values.

Hematologic parameters	Categories		Unadjusted OR (95% CI)	*p*	Multivariable regression adjusted OR (95% CI)	*p*	PSM adjusted OR (95% CI)	*p*
Blood glucose	Continuous	Per SD	1.27 (1.21–1.33)	<0.001	1.21 (1.13–2.28)	<0.001	NA	NA
	Clinical cutoffs	<6.10 mmol/L	1 [Reference]	<0.001	1 [Reference]	<0.001	1 [Reference]	<0.001
		≥6.10 mmol/L	3.88 (2.87–5.25)		2.46 (1.74–3.47)		2.14 (1.50–3.05)	
	Quartile	Q1 (4.00–5.30)	1 [Reference]	NA	1 [Reference]	NA	1 [Reference]	NA*
		Q2 (5.30–6.10)	2.17 (1.30–3.63)	0.003	1.90 (1.07–3.34)	0.028	1.20 (0.37–3.89)	0.764
		Q3 (6.10–7.40)	4.36 (2.67–7.13)	<0.001	1.69 (1.29–2.22)	<0.001	2.12 (1.22–3.68)	0.008
		Q4 (≥7.40)	8.56 (5.66–13.73)	<0.001	1.71 (1.41–2.07)	<0.001	3.55 (1.93–6.50)	<0.001
ALB	Continuous	Per SD	0.91 (0.88–0.94)	<0.001	0.94 (0.81–0.98)	0.001	NA	NA
	Clinical cutoffs	≥35 g/L	1 [Reference]	<0.001	1 [Reference]	0.010	1 [Reference]	0.006
		<35 g/L	2.00 (1.30–3.08)		1.59 (1.11–2.27)		1.75 (1.18–2.63)	
	Quartile	Q1 (<35)	1 [Reference]	NA	1 [Reference]	NA	1 [Reference]	NA
		Q2 (35–38)	0.76 (0.53–1.09)	0.131	0.68 (0.45–1.02)	0.062	0.80 (0.51–1.27)	0.344
		Q3 (38–41)	0.52 (0.36–0.75)	0.001	0.82 (0.66–1.03)	0.094	0.51 (0.32–0.82)	0.006
		Q4 (≥41)	0.31 (0.21–0.46)	<0.001	0.78 (0.65–0.92)	0.004	0.32 (0.19–0.55)	<0.001
GLB	Continuous	Per SD	1.08 (1.05–1.11)	<0.001	1.08 (1.04–1.11)	0.001	NA	NA
	Clinical cutoffs	≤30 g/L	1 [Reference]	0.003	1 [Reference]	<0.001	1 [Reference]	0.074
		>30 g/L	1.65 (1.19–2.29)		4.02 (2.62–6.18)		1.38 (0.97–1.97)	
	Quartile	Q1 (<25)	1 [Reference]	NA	1 [Reference]	NA	1 [Reference]	NA
		Q2 (25–27)	1.39 (0.93–2.06)	0.106	1.77 (1.08–2.82)	0.023	1.83 (1.05–3.21)	0.034
		Q3 (28–31)	1.58 (1.06–2.37)	0.026	1.26 (0.99–1.59)	0.057	1.45 (1.10–1.92)	0.009
		Q4 (≥31)	2.24 (1.54–3.26)	<0.001	1.35 (1.16–1.57)	<0.001	1.40 (1.18–1.66)	<0.001
HDL	Continuous	Per SD	0.53 (0.36–0.77)	0.001	0.67 (0.46–0.98)	0.038	NA	NA
	Clinical cutoffs	≥1.00 mmol/L	1 [Reference]	0.009	1 [Reference]	0.300	1 [Reference]	0.322
		<1.00 mmol/L	1.47 (1.10–1.96)		1.19 (0.85–1.67)		1.19 (0.84–1.69)	
	Quartile	Q1 (<0.99)	1 [Reference]	NA	1 [Reference]	NA	1 [Reference]	NA
		Q2 (0.99–1.21)	0.84 (0.59–1.21)	0.035	0.98 (0.65–1.49)	0.940	0.72 (0.43–1.21)	0.208
		Q3 (1.21–1.47)	0.69 (0.48–0.99)	0.049	0.92 (0.74–1.14)	0.423	0.74 (0.45–1.22)	0.237
		Q4 (≥1.47)	0.50 (0.34–0.73)	<0.001	0.84 (0.72–0.97)	0.021	0.49 (0.29–0.81)	0.005

BG, Blood glucose; ALB, Albumin; GLB, Globulin; HDL, High-density lipoprotein; SD, standard deviation; NA, not available; OR, odds ratio; PSM, propensity scores matching.

Our analysis showed that hyperglycemia, hypoalbuminemia, hyperglobulinemia, and low HDL were associated with an increased risk of UTIs. After multivariate regression analysis, compared to patients in group Q1, patients in groups Q3 (Blood glucose: OR 1.69, *p* < 0.001; ALB: OR 0.82, *p* = 0.094; GLB: OR 1.26, *p* = 0.057), Q4 (Blood glucose: OR 1.71, *p* < 0.001; ALB: OR 0.78, *p* = 0.004; GLB: OR 1.35, *p* < 0.001; HDL: OR 0.84, *p* = 0.021) had a significantly increased risk of UTIs. After adjustment with propensity score matching, compared to patients in group Q3 (Blood glucose: OR 2.12, *p* = 0.008; ALB: OR 0.51, *p* = 0.006; GLB: OR 1.45, *p* = 0.009), and group Q4 (Blood glucose: OR 3.55, *p* < 0.001; ALB: OR 0.32, *p* < 0.001; GLB: OR 1.40, *p* < 0.001; HDL: OR 0.49, *p* = 0.005) also had a significantly increased risk of UTIs (Table 2).

**Figure 2:**
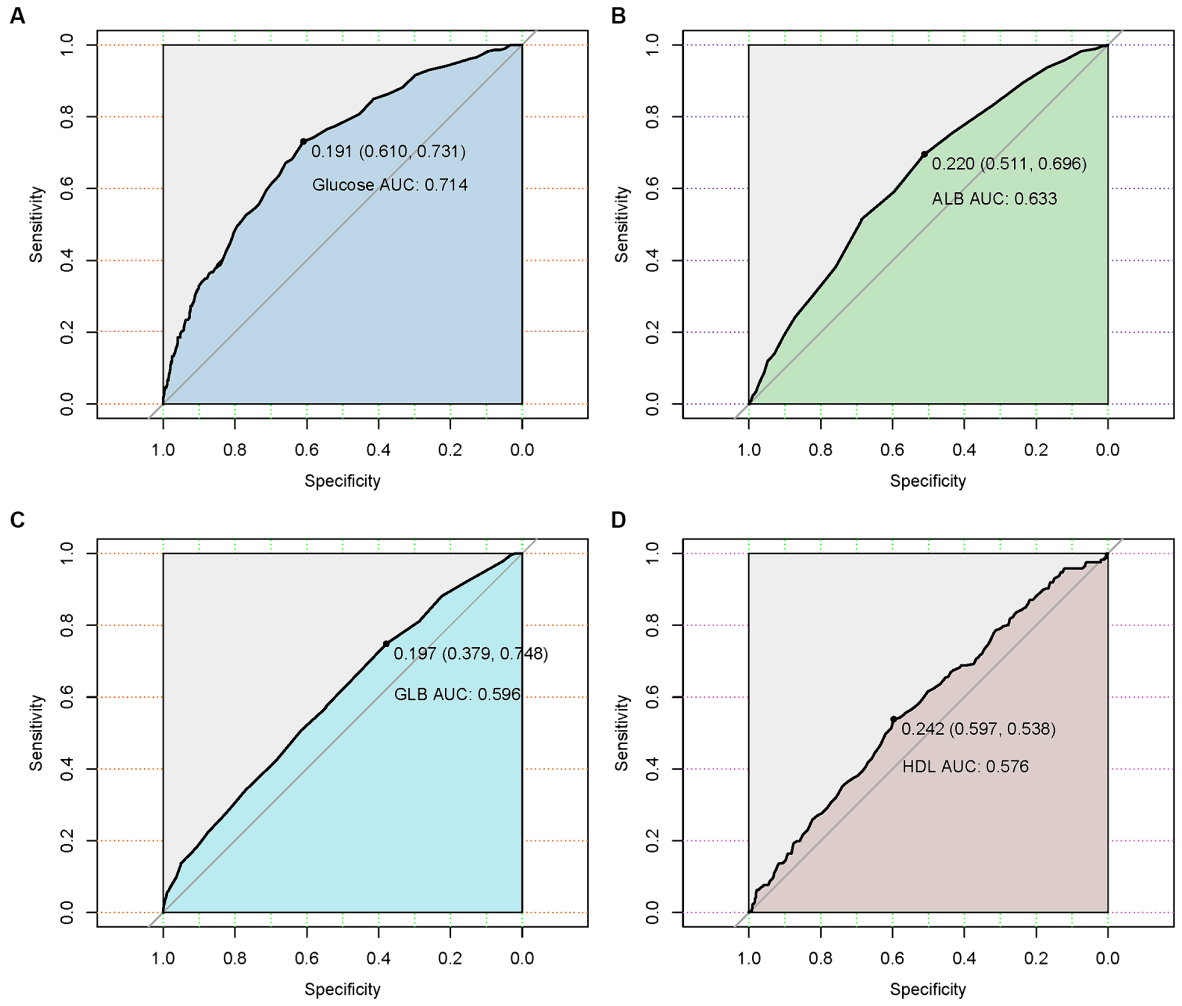
Receiver operating characteristic (ROC) curves of four hematological indicators (blood glucose, albumin, globulin, and high-density lipoprotein) predicting the presence of urinary tract infection in geriatric patients with hip fractures. **(A)** The ROC curve of blood glucose. **(B)** The ROC curve of albumin. **(C)** The ROC curve of globulin. (D) The ROC curve of blood glucose and high-density lipoprotein.

**Figure 3:**
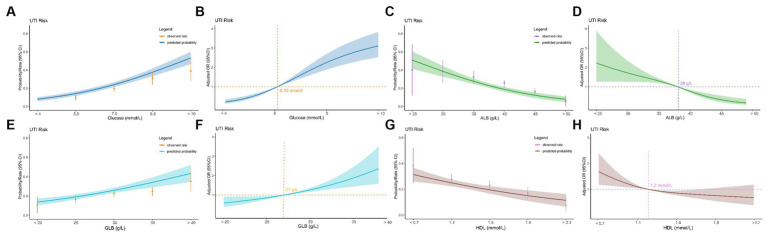
Relationship between four hematological indicators (blood glucose, albumin, globulin, and high-density lipoprotein) and UTIs in geriatric patients with hip fractures. **(A)** Predicted probabilities and the observed rate of UTIs (blood glucose). **(B)** Adjusted odds ratios (ORs) and 95% confidence intervals (CIs) are shown for each 1.5 mmol/L deviations away from the reference value (blood glucosse). **(C)** Predicted probabilities and the observed rate of UTIs (albumin). **(D)** Adjusted odds ratios (ORs) and 95% confidence intervals (CIs) are shown for each 5.0 g/L deviations away from the reference value (albumin). **(E)** Predicted probabilities and the observed rate of UTIs (globulin). **(F)** Adjusted odds ratios (ORs) and 95% confidence intervals (CIs) are shown for each 5.0 mmol/L deviations away from the reference value (globulin). **(G)** Predicted probabilities and the observed rate of UTIs (high-density lipoprotein). **(H)** Adjusted odds ratios (ORs) and 95% confidence intervals (CIs) are shown for each 0.4 mmol/L deviations away from the reference value (high-density lipoprotein).

ROC curve analysis assessed the ability of hematologic parameters to predict UTIs. Glucose (AUC 0.714), albumin (AUC 0.633), globulin (AUC 0.596) and HDL (AUC 0.576) showed moderate predictive value (Table 3 and Figure 2). The adjusted odds ratios and 95% confidence intervals demonstrate significant associations between concentrations of blood glucose, albumin, globulin, and high-density lipoprotein and the risk of urinary tract infection. Additionally, the dose–response relationships between blood glucose, albumin, globulin, high-density lipoprotein and the occurrence of UTIs (Figure 3).

**Table 3:** Model performance of individual hematological indicators for predicting UTIs.

Variables	AUC (95% CI)	ACC (%, 95 CI)	SEN (%, 95 CI)	SPE (%, 95 CI)	PPV (%, 95 CI)	NPV (%, 95 CI)
Blood glucose	0.714 (0.680–0.747)	63.8 (63.7–63.8)	73.1 (67.9–78.2)	61.0 (57.8–64.1)	36.2 (32.2–40.1)	88.2 (85.7–90.7)
ALB	0.633 (0.597–0.669)	55.4 (55.4–55.4)	69.6 (64.2–74.9)	51.1 (47.9–54.3)	30.1 (26.6–33.6)	84.7 (81.8–87.7)
GLB	0.596 (0.559–0.636)	(46.5 46.4–46.5)	74.8 (69.8–79.9)	37.9 (34.8–41.0)	26.7 (23.7–29.8)	83.3 (79.7–86.8)
HDL	0.576 (0.539–0.613)	58.3 (58.3–58.4)	53.8 (48.1–59.6)	59.7 (56.6–62.8)	28.8 (24.9–32.6)	80.1 (78.1–83.9)

AUC, area under curve; ACC, accuracy; SEN, sensitivity; SPE, specificity; PPV, positive predictive value; NPV, negative predictive value.

We conducted additional analyses to separately evaluate potential interactions between hyperglycemia, hypoalbuminemia, hyperglobulinemia, and low HDL with other variables (Supplementary Figures 2–5). We observed a significant interaction between hypoalbuminemia and treatment type (interaction *p*<0.001), bedridden time (interaction *p* = 0.015), and ASA (interaction *p* = 0.008), indicating that the effect of hypoalbuminemia on the risk of UTIs may differ depending on treatment type, bedridden time, and ASA. The subgroup analyses showed that the patterns of association of hyperglycemia, hyperglobulinemia, and low HDL with urinary tract infection were consistent across different populations.

## Discussion

Urinary tract infection (UTI) is a common complication in elderly patients with hip fractures, which can significantly affect their rehabilitation and prognosis (2, 7). The pathogenesis of UTI in these patients is multifactorial. Invasive procedures like catheterization during surgery increase the risk of bacterial inoculation into the urinary tract (18). Furthermore, urinary obstruction and incomplete bladder emptying allow bacterial colonization and overgrowth, predisposing to infection (4). The geriatric patients also have decreased immunity and frequent comorbidities, making them vulnerable to UTIs (19, 20). Timely diagnosis and management of UTIs are thus critical in elderly hip fracture patients to optimize their outcomes.

To investigate the prediction of UTI risk, we investigated the predictive value of common hematological and biochemical parameters UTIs in a large cohort of elderly hip fracture patients. In this retrospective study of 1,231 geriatric hip fracture patients, we found that hyperglycemia (>6.10 mmol/L), hypoalbuminemia (<35 g/L), Hyperglobulinemia (>30 g/L), and lower HDL cholesterol levels (<1.00 mmol/L) were associated with increased odds of urinary tract infections (UTIs). After multivariate analysis and propensity score matching, the results remained robust except for high-density lipoprotein. These findings suggest common hematological and biochemical parameters could serve as useful predictors for identifying patients prone to develop UTIs in this vulnerable population.

### Blood glucose

This study has identified hyperglycemia (>6.10 mmol/L) as an independent risk factor for urinary tract infections (UTIs) in geriatric patients with hip fractures. The multivariable analysis demonstrated significantly higher odds of UTIs in the presence of hyperglycemia (OR 2.46, 95%CI 1.74–3.47). Our research results are consistent with previous research findings. Yao et al. found patients with admission blood glucose >10.00 mmol/L had a significantly higher risk of CAUTIs than those with 4.00–6.09 mmol/L (OR 3.10, 95%CI 1.65–5.82), and the relationship between admission hyperglycemia and CUUTIs was even closer, patients with >10.00 mmol/L had a markedly higher risk of CUUTIs than those with 4.00–6.09 mmol/L (OR 4.42, 95%CI 2.09–9.33) (3). Additionally, Kobayashi et al. found significant associations between predisposition to UTI and HbA1c level or duration of diabetes (21). Numerous studies found that the risk of urinary tract infection (UTI) is higher in diabetics compared to non-diabetics (22, 23). However, these studies did not specifically investigate the association between admission hyperglycemia and UTIs.

Several mechanisms may contribute to this association in this susceptible population. Hyperglycemia causes osmotic diuresis and polyuria, which can lead to incomplete bladder emptying and urinary stasis (24). This allows bacterial overgrowth in residual urine. Hyperglycemia also impairs neutrophil function and weakens the immune response to pathogens (25). The acidic urine environment further encourages bacterial proliferation. At the cellular level, disrupts the antibacterial glycosaminoglycan layer lining the bladder (26).

### Albumin

Our findings demonstrate hypoalbuminemia to be an independent predictor of urinary tract infections (UTIs) among geriatric patients with hip fractures. Several physiologic mechanisms may underlie this association between low serum albumin levels and heightened UTI risk. Albumin plays a vital role in humoral immunity and defense against pathogens (27). Through exerting antioxidative effects and binding bacteria, toxins, and other infectious particles, albumin protects against invading microorganisms (27, 28). Hypoalbuminemia diminishes these functions, thereby compromising immune defenses and increasing susceptibility to infections including UTIs (21). Furthermore, albumin helps maintain proper oncotic pressure, preventing fluid extravasation that could impede urine flow and heighten the risk of UTIs (29). Albumin also impacts pharmacokinetics by binding drugs and affecting their distribution and elimination (30). Hypoalbuminemia may increase the unbound antibiotic fraction, reducing therapeutic efficacy against urinary pathogens.

Hypoalbuminemia has been found to be a significant risk factor for urinary tract infections (UTIs) in various clinical settings. In a large retrospective patient population study, Yoshida et al. found pre-operative hypoalbuminemia was found to be a significant risk factor for UTIs after lumbar spine surgery (31). Another study found that hypoalbuminemia was associated with increased 30-day complications following rectourethral fistula repair, including UTIs (32). Our findings reinforce the prognostic value of hypoalbuminemia specifically for predicting post-operative UTIs in elderly patients with hip fractures. Routine albumin levels can thus help guide preventive efforts in this high-risk population.

We observed significant interactions of albumin with treatment type, bedridden time, and ASA score in relation to UTI risk, suggesting albumin’s effect may differ based on surgery trauma, immobility duration, and health status. These interactions indicate the need for stratified analysis to guide personalized UTI prevention strategies accounting for influences on albumin’s predictive utility. However, there is currently no literature specifically studying the interaction between albumin and treatment type, bed rest time, and ASA score. Previous studies have cautioned that performing multiple subgroup analyses can increase the chance of false positive findings (33). Therefore, further research should explore potential mechanisms underlying albumin’s interactions with these factors and vulnerability to UTIs.

### Globulin

We found urinary tract infections were significantly more likely in patients with serum globulin levels >30 g/L compared to those with levels ≤30 g/L. Although less studied than albumin, high globulin may also indicate poorer immune function and higher urinary tract infection risk (34). Elevated globulins can reflect chronic inflammation, autoimmune conditions, or chronic infections which could predispose patients to further infections (35). Proposed mechanisms include antibody production driven by immune stimulation and impaired cell-mediated immunity (36). Small studies linked hyperglobulinemia to increased post-operative infections in certain surgeries (37). Our findings suggest this relationship may generalize to geriatric hip fracture patients as well.

However, effects were weaker for globulin than albumin in our study. Globulin has a less well-established role as a prognostic nutritional marker compared to albumin. The serum albumin/globulin ratio may have greater predictive utility than globulin alone (38). Optimal cut-off values are also less certain, as hyperglobulinemia definitions range from >30 to >40 g/L across studies (39). Additional research is warranted to elucidate the mechanisms linking globulin with infection risk and determine clinically useful cut-off values and standardized ratios. Nonetheless, our study provides initial evidence that high globulin levels may help identify geriatric hip fracture patients at increased risk of UTIs post-operatively. This could allow closer monitoring and preventive efforts in high-risk patients to reduce morbidity. Further investigation of globulin’s utility for infection risk stratification is warranted in this susceptible population.

### High-density lipoprotein

The univariate analysis showed that low HDL (<1.0 mmol/L) was associated with significantly higher odds of UTI compared to HDL ≥1.0 mmol/L. This aligns with some prior studies finding a relationship between low HDL and infection risk. A study published in the European Heart Journal found that both low and high concentrations of HDL cholesterol were associated with a high risk of infectious disease, including urinary tract infections, in the general population (40). A population-based cohort study found that low levels of HDL cholesterol were associated with increased morbidity and mortality from infectious diseases, including UTIs (41). Proposed reasons include HDL’s roles in binding and neutralizing bacterial lipopolysaccharides, as well as modulating cytokine production and leukocyte function (40).

However, after adjusting for confounders in the multivariable regression model in our study, the association between low HDL and UTI was no longer statistically significant. The adjusted odds ratio was 1.19 (95% CI 0.84–1.69, *p* = 0.322), indicating HDL was not an independent predictor of UTI when accounting for other factors.

The attenuation of effect after multivariate adjustment implies the univariate relationship was likely driven by confounding rather than any direct predictive ability of HDL for UTI (42). Particularly, many comorbid conditions that elevate UTI risk like diabetes and kidney disease are also associated with lower HDL levels (43, 44). Overall, current evidence does not strongly support a predictive or causal role of HDL in UTIs among geriatric hip fracture patients. Prospective studies are needed to clarify whether an association between low HDL and UTIs.

### Limitations

Our study has several strengths, including a relatively large sample size, and comprehensive clinical data to control for confounders. Focusing on commonly available hematological and biochemical parameters enhances real-world applicability. Leveraging routine blood tests to predict postoperative infections allows early identification of high-risk patients requiring preventive interventions. Overall, it provides an effective clinical decision tool to enable precision targeting of UTI prevention efforts in elderly hip fracture populations.

However, it is important to acknowledge the limitations of this study. First, this single-center retrospective study is subject to inherent selection and information bias. Future multi-center prospective cohort studies will help validate the generalizability of our findings. Second, the predictive performance of hematological and biochemical parameters for UTI in our study was only moderate, with AUC values around 0.6. In clinical application, these parameters should be interpreted in combination with other patient factors. Third, we did not perform subgroup analyses by UTI type. Future research should examine the predictors in different UTIs such as catheter-associated and catheter-unassociated UTIs. Fourth, the retrospective design precluded dynamic monitoring. The value of temporal changes in these markers for prognostication and treatment response requires future prospective assessment. Finally, although propensity score matching was used to adjust for confounders, the sample size was relatively small after matching and some subgroup samples were limited. This may affect result robustness.

## Conclusion

In conclusion, our study demonstrates that glucose, albumin, and globulin levels are useful hematological and biochemical parameters for identifying geriatric hip fracture patients at high risk of UTIs. These inexpensive and common hematological and biochemical parameters could assist in guiding preventive efforts to reduce UTIs and improve outcomes for vulnerable elderly patients undergoing hip fracture surgery.

## Data availability statement

All the data used and analyzed during the current study are available from the corresponding author upon reasonable request.

## Ethics statement

Written informed consent was not obtained from the individual(s) for the publication of any potentially identifiable images or data included in this article because this is just retrospective study analyzing existing medical data, informed consent from the patients was not required. The hematological data and information on urinary tract infections had already been collected during the course of routine clinical care and were obtained through a review of medical records. No additional interventions were performed and the analysis relied only on these previously recorded clinical variables. Patient identity remained anonymized throughout the study. The study procedures were reviewed and approved by the Institutional Review Board of Dandong Central Hospital, who determined that individual informed consent was not necessary for this type of retrospective chart review that posed minimal risk to patients. This approach aligns with ethical guidelines for medical research involving human subjects when utilizing already collected data where consent would be impractical to obtain. By waiving the informed consent requirement, we were able to conduct this research efficiently while upholding patient privacy and confidentiality.

## Author contributions

WT: Writing – original draft, Writing – review & editing. WY: Writing – review & editing, Software. WW: Writing – review & editing. QL: Writing – original draft. WD: Writing – review & editing. RH: Writing – original draft.

## Glossary

UTI: Urinary tract infection
BG: Blood glucose
ALB: Albumin
GLB: Globulin
HDL: High-density lipoprotein
ROC: Receiver operating characteristic curve
STROCS: Strengthening the Reporting of Cohort Studies in Surgery
IQR: Interquartile range
AUC: Area under curve
ACC: Accuracy
SEN: Sensitivity
SPE: Specificity
PPV: Positive predictive value
NPV: Negative predictive value
OR: Odds ratios
CI: Confidence intervals
SE: Standard error
CT: Computed Tomography
MRI: Magnetic Resonance Imaging
NA: Not available
