# Supplementary material for: Common hematological and biochemical parameters for predicting urinary tract infections in geriatric patients with hip fractures
Source: Front Med (Lausanne). 2024 May 30;11:1333472. doi: 10.3389/fmed.2024.1333472 (PMC11169829; doi:10.3389/fmed.2024.1333472)
Supplement: Supplementary file 1 [file Data_Sheet_1.docx]

**Appendix:**

**eFigure 1 Flow diagram of patients included in the study.**

**eFigure 2 Subgroup analysis of adjustment association between glucose and UTIs after propensity score matching.**

**eFigure 3 Subgroup analysis of adjustment association between ALB and UTIs after propensity score matching.**

**eFigure 4 Subgroup analysis of adjustment association between GLB and UTIs after propensity score matching.**

**eFigure 5 Subgroup analysis of adjustment association between HDL and UTIs after propensity score matching.**

**eTable 1 Comparison of the incidence of UTIs before and after PSM based on** **hematological indicators.**

**eTable2 Univariate and multivariate regression analyses of risk factors for UTIs (Glucose -UTIs)**

**eTable3 Patient characteristics before and after propensity score matching by glucose clinical cutoffs (Glucose-UTIs)**

**eTable4 Univariate and multivariate regression analyses of risk factors for UTIs (ALB-UTIs)**

**eTable5 Patient characteristics before and after propensity score matching by ALB Clinical cutoffs (ALB-UTIs)**

**eTable6 Univariate and multivariate regression analyses of risk factors for UTIs (GLB-UTIs)**

**eTable7 Patient characteristics before and after propensity score matching by GLB Clinical cutoffs (GLB-UTIs)**

**eTable8 Univariate and multivariate regression analyses of risk factors for UTIs (HDL-UTIs)**

**eTable9 Patient characteristics before and after propensity score matching by HDL Clinical cutoffs(HDL-UTIs)**

**
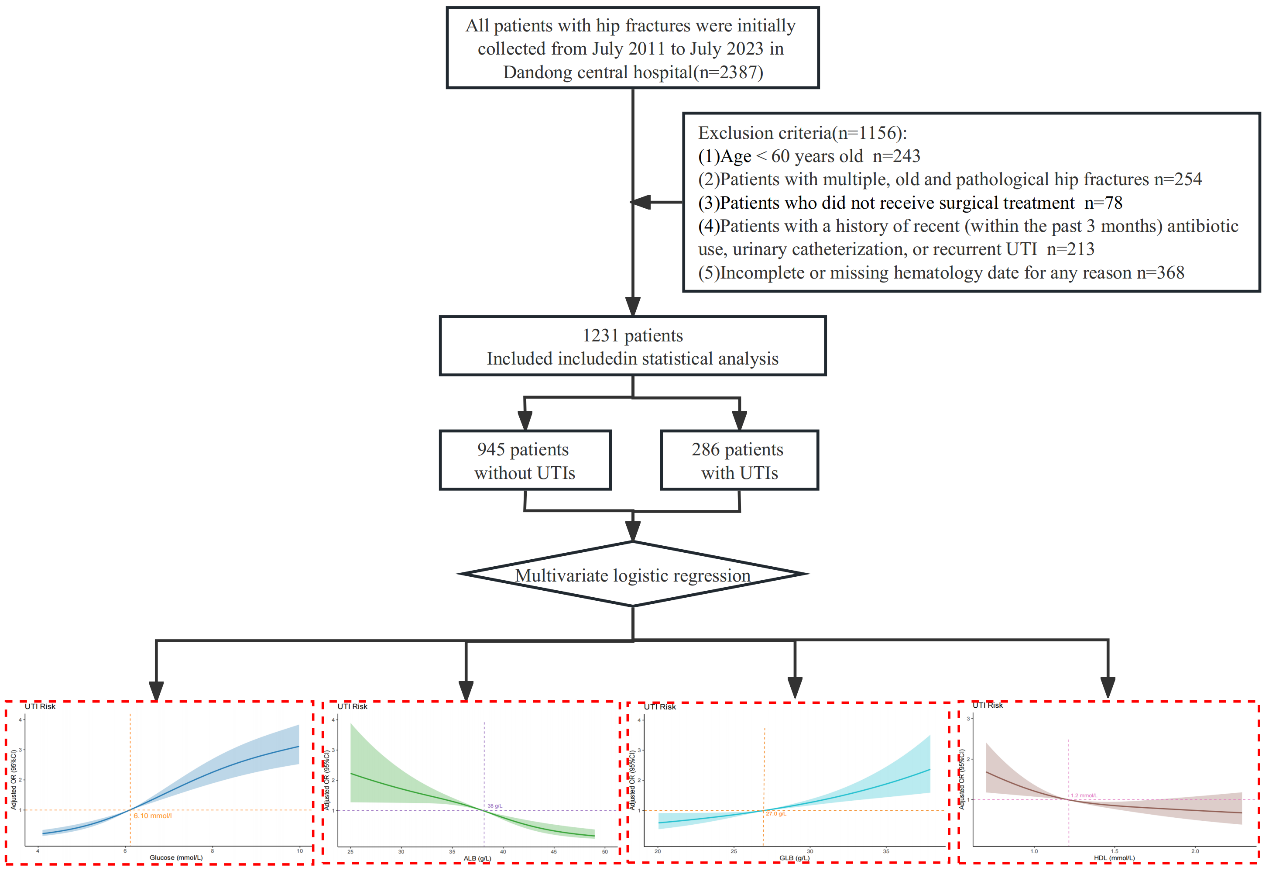
**

**eFigure 1 Flow diagram of patients included in the study.**


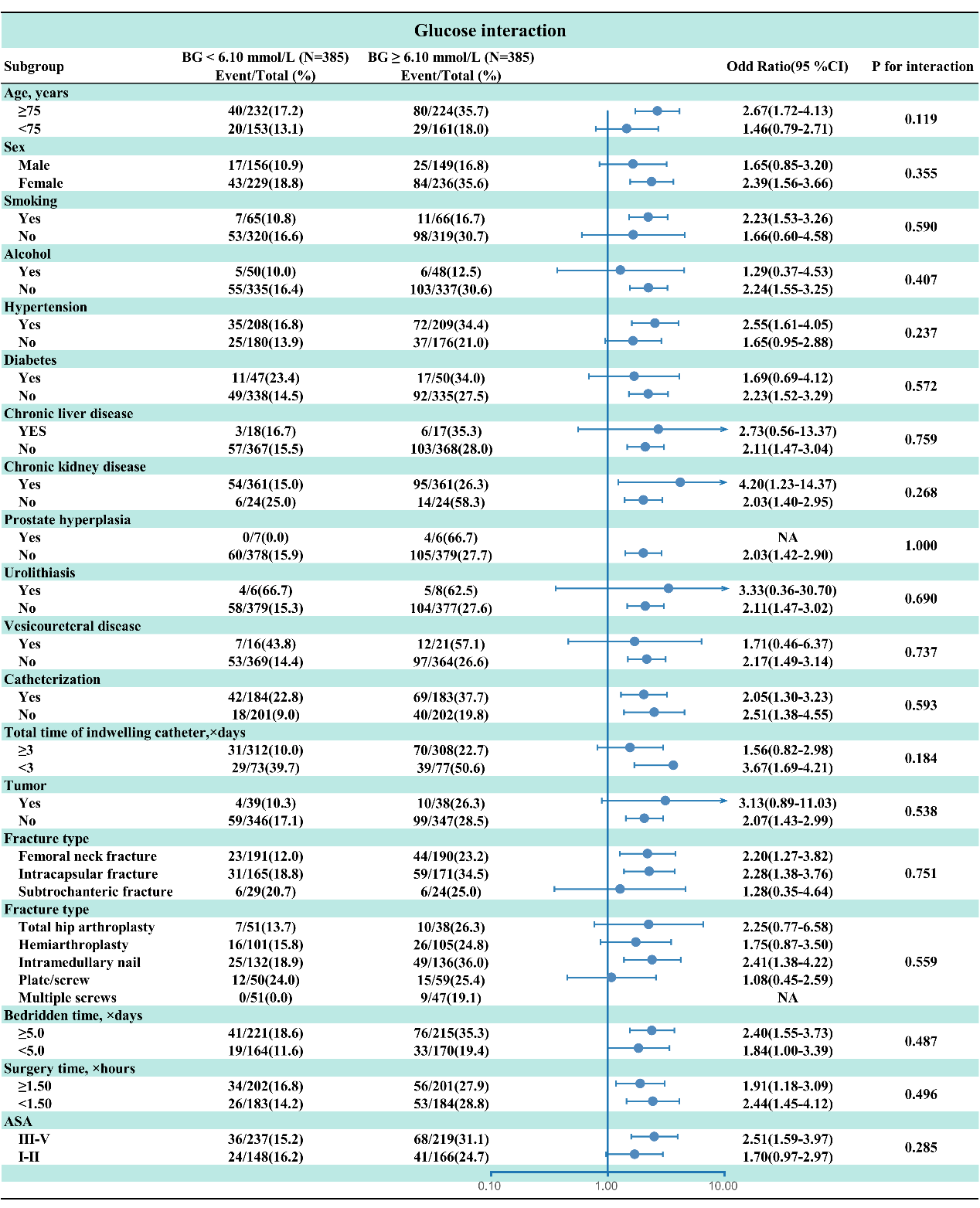


**eFigure 2 Subgroup analysis of adjustment association between glucose and UTIs a after propensity score matching**

**
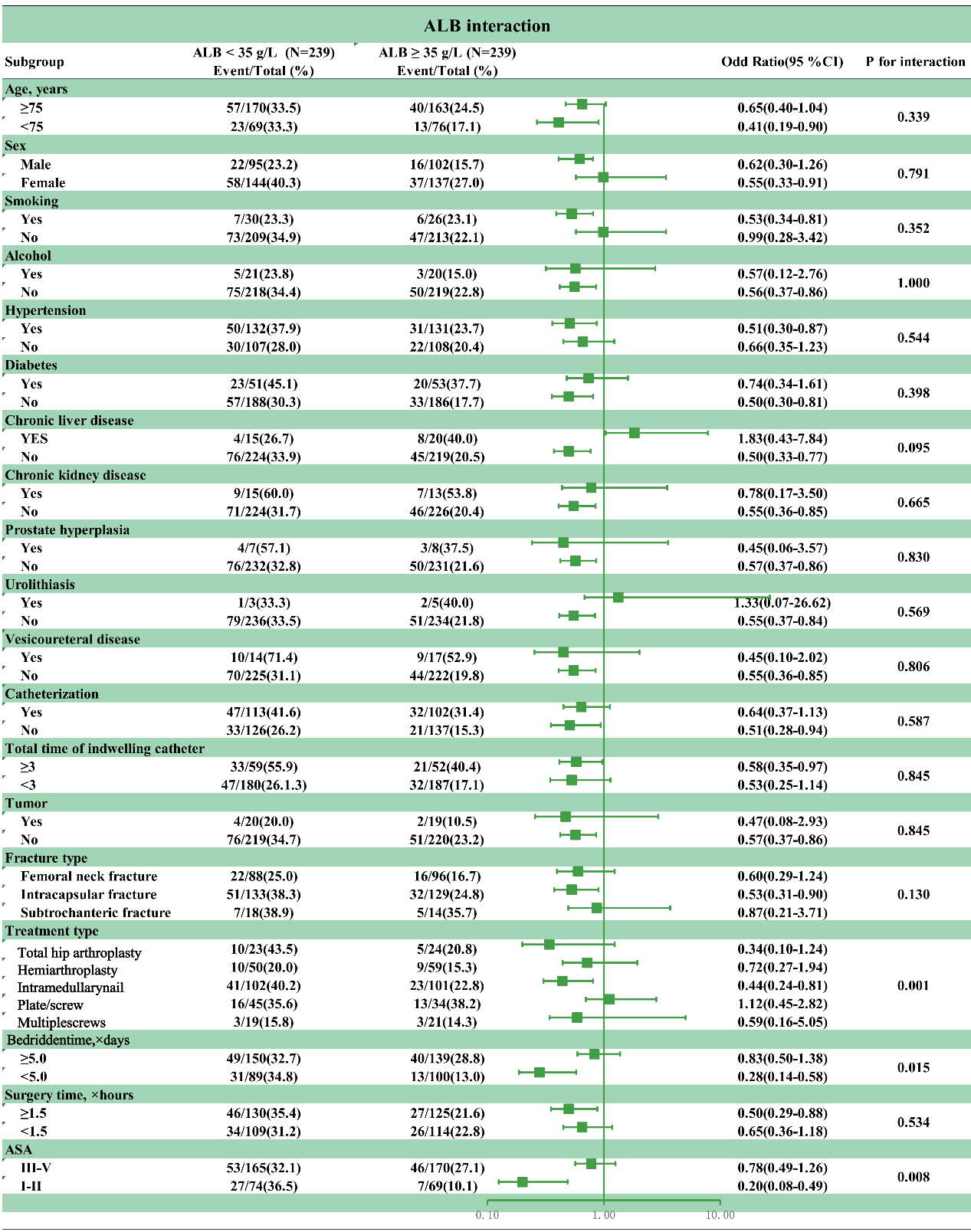
**

**eFigure 3 Subgroup analysis of adjustment association between ALB and UTIs a after propensity score matching.**

**
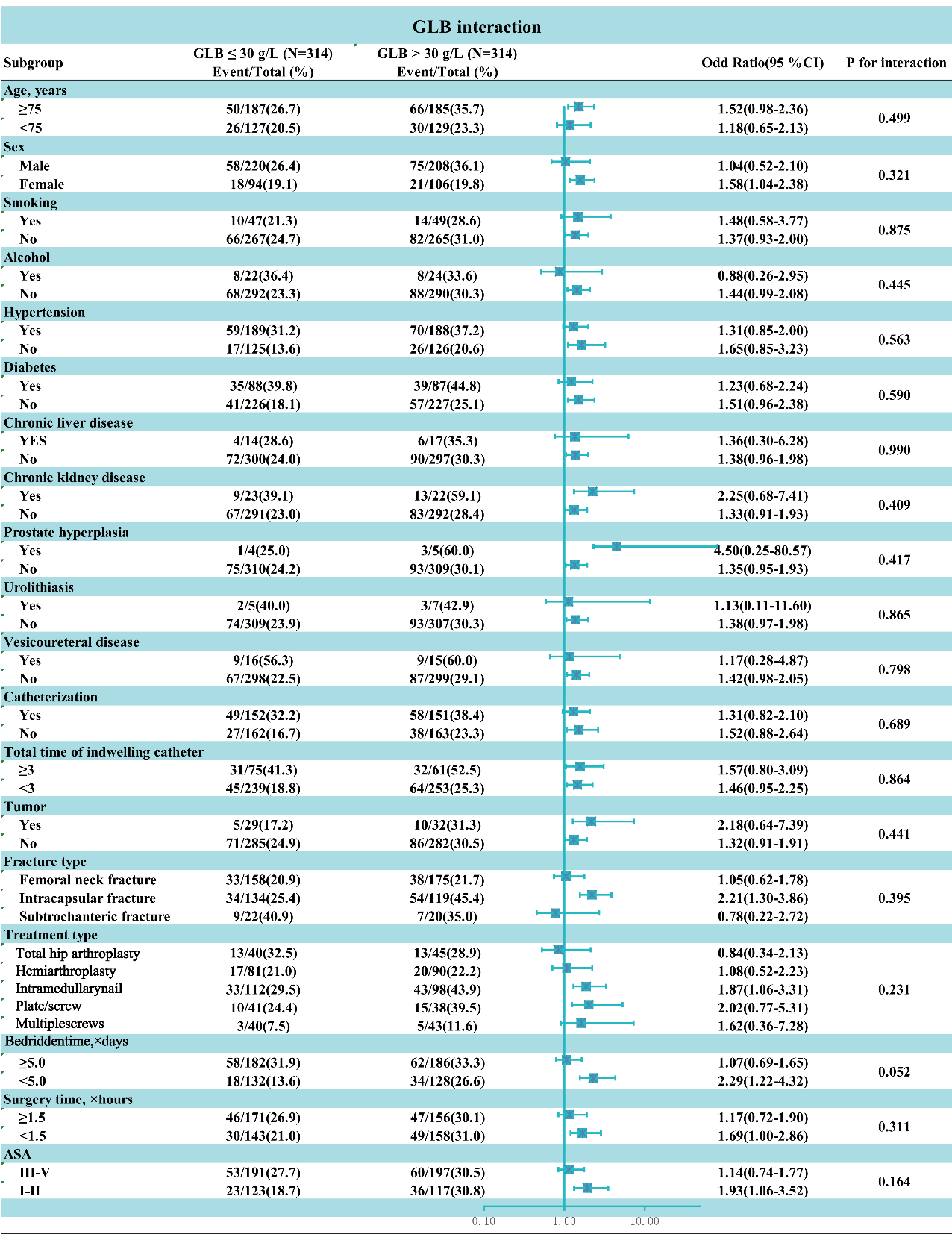
eFigure 4 Subgroup analysis of adjustment association between GLB and UTIs a after propensity score matching.**


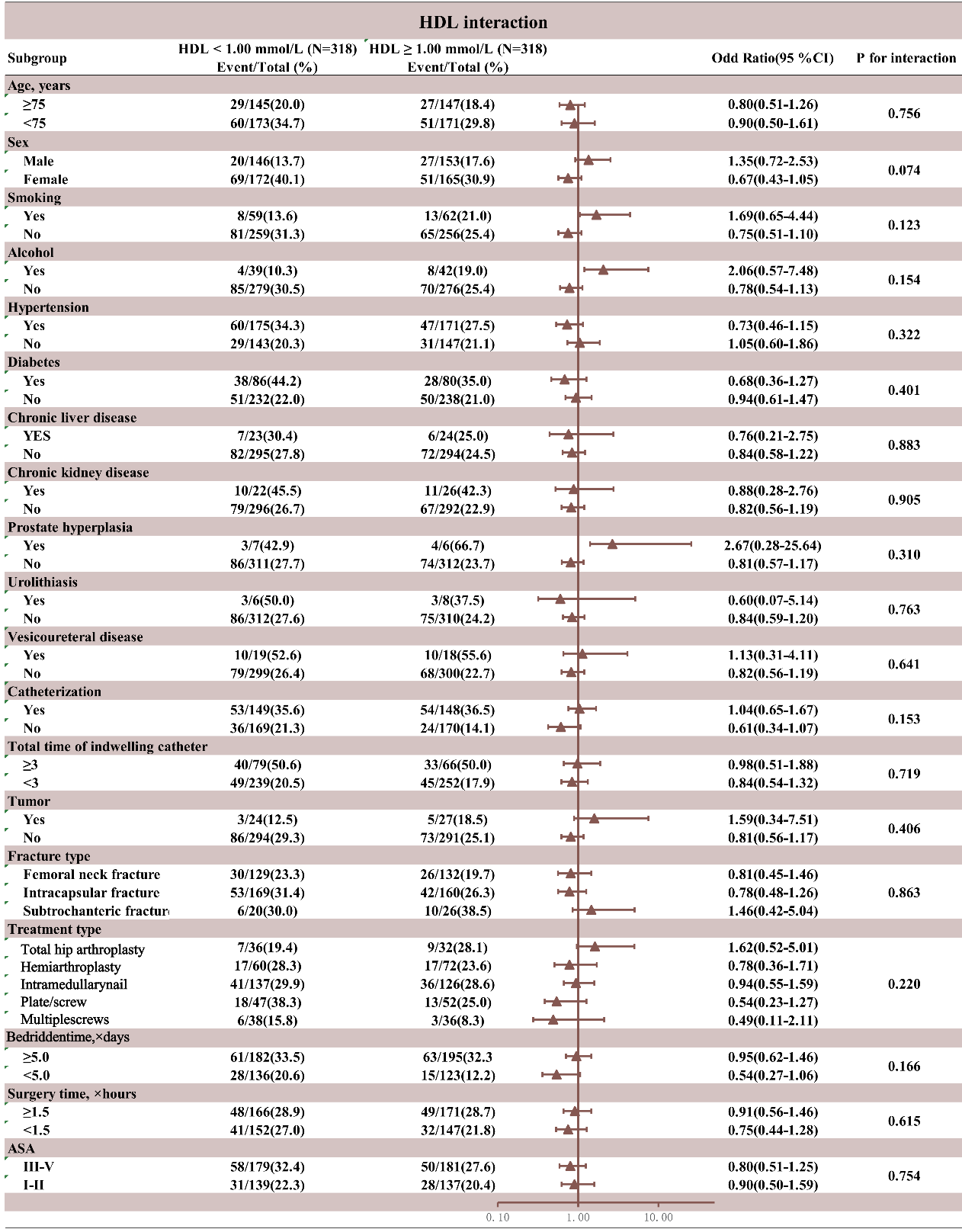


**eFigure 5 Subgroup analysis of adjustment association between HDL and UTIs a after propensity score matching.**

**eTable 1** **Comparison of the incidence of UTIs before and after PSM based on hematological indicators.**

| Hematologic parameters | No. (%)  Clinical  cutoffs | Before PSM | | p | After PSM | | p |
| --- | --- | --- | --- | --- | --- | --- | --- |
|  |  | Non-UTIs | UTIs |  | Non-UTIs | UTIs |  |
| Glucose | < 6.10 mmol/L | 513(54.3) | 67(15.4) | <0.001 | 325(51.6) | 60(42.9) | <0.001 |
|  | ≥ 6.10 mmol/L | 432(47.7) | 219(84.6) |  | 305(48.4) | 80(57.1) |  |
| ALB | ≥ 35 g/L | 775(65.9) | 199(69.6) | <0.001 | 186(53.9) | 53(39.8) | 0.006 |
|  | < 35 g/L | 170(34.1) | 87(30.4) |  | 159(46.1) | 80(60.2) |  |
| GLB | ≤ 30 g/L | 724(76.6) | 187(65.4) | <0.001 | 238(52.2) | 76(44.2) | 0.074 |
|  | > 30 g/L | 221(23.4) | 99(34.6) |  | 218(47.8) | 96(55.8) |  |
| HDL | ≥ 1.00 mmol/L | 715(75.7) | 194(67.8) | 0.008 | 240(51.2) | 78(46.7) | 0.322 |
|  | < 1.00 mmol/L | 230(24.3) | 92(32.2) |  | 229(48.8) | 89(53.3) |  |

Abbreviations: ALB, Albumin; GLB, Globulin; HDL, High-density lipoprotein; p for trend.

**eTable2 Univariate and multivariate regression analyses of risk factors for UTIs (Glucose -UTIs)**

| Characteristics | Univariate | | | Multivariate | | |
| --- | --- | --- | --- | --- | --- | --- |
|  | OR | 95%CI | P-value | OR | 95%CI | P-value |
| Age | 1.05 | 1.03-1.06 | <0.001 | 1.03 | 1.01-1.05 | 0.004 |
| Female gender | 2.21 | 1.65-2.97 | <0.001 | 2.48 | 2.48-3.70 | <0.001 |
| Smoking | 0.57 | 0.38-0.85 | 0.006 | 0.92 | 0.61-1.90 | 0.807 |
| Alcohol | 0.50 | 0.31-0.82 | 0.006 | 0.82 | 0.45-1.87 | 0.818 |
| Hypertension | 2.06 | 1.57-2.71 | <0.001 | 1.33 | 0.96-1.85 | 0.092 |
| Diabetes | 1.98 | 2.23-3.97 | <0.001 | 1.35 | 0.91-1.99 | 0.136 |
| Chronic liver disease | 1.56 | 0.88-2.77 | 0.13 | <NA> | <NA> | <NA> |
| Chronic kidney disease | 3.36 | 2.02-5.60 | <0.001 | 2.28 | 1.19-4.38 | 0.013 |
| Prostate hyperplasia | 2.72 | 1.26-5.87 | 0.01 | 4.57 | 1.78-11.72 | 0.002 |
| Urolithiasis | 2.69 | 1.05-6.88 | 0.04 | 2.04 | 0.60-7.00 | 0.255 |
| Vesicoureteral disease | 3.98 | 2.33-6.824 | <0.001 | 1.93 | 0395-3.94 | 0.071 |
| Catheterization | 2.28 | 1.74-3.00 | <0.001 | 1.16 | 0.78-1.72 | 0.462 |
| Total time of indwelling catheter | 1.21 | 1.16-1.26 | <0.001 | 1.17 | 1.10-1.24 | <0.001 |
| Tumor | 0.87 | 0.52-1.36 | 0.479 | <NA> | <NA> | <NA> |
| Fracture type | 0.63 | 0.507-0.775 | <0.001 | 0.55 | 0.41-0.75 | <0.001 |
| Treatment Type | 0.88 | 0.79-0.98 | 0.02 | 0.86 | 0.73-1.02 | 0.085 |
| Bedridden time | 1.08 | 1.05-1.12 | <0.001 | 1.04 | 1.01-1.08 | 0.023 |
| Surgery time | 1.17 | 1.00-1.36 | 0.054 | <NA> | <NA> | <NA> |
| ASA | 1.46 | 1.11-1.92 | 0.006 | 1.43 | 1.02-2.01 | 0.039 |
| Glucose（≥ 6.10 mmol/L） | 3.88 | 2.87-5.25 | <0.001 | 2.46 | 1.74-3.47 | <0.001 |

**eTable3 Patient characteristics before and after propensity score matching by glucose clinical cutoffs (Glucose-UTIs)**

| Characteristics | Before matching | | | After matching | | |
| --- | --- | --- | --- | --- | --- | --- |
|  | Glucose＜6.10  (n=580) | Glucose≥ 6.10  (n=651) | SMD | Glucose＜6.10  (n=385) | Glucoese ≥ 6.10  (n=385) | SMD |
| Demographic |  |  |  |  |  |  |
| Age, × years (Mean, SD) | 72.77 (9.83) | 76.67 (9.00) | 0.414 | 76.01 (9.50) | 75.67 (9.21) | 0.036 |
| Female gender (n,%) | 323 (55.7) | 422 (64.8) | 0.187 | 229 (59.5) | 236 (61.3) | 0.037 |
| Smoking (n,%) | 112 (19.3) | 97 (14.9) | 0.117 | 65 (16.9) | 66 (17.1) | 0.007 |
| Alcohol (n,%) | 73 (12.6) | 70 (10.8) | 0.057 | 50 (13.0) | 48 (12.5) | 0.016 |
| Comorbidities |  |  |  |  |  |  |
| Hypertension (n,%) | 238 (41.0) | 387 (59.4) | 0.374 | 205 (53.2) | 209 (54.3) | 0.021 |
| Diabetes (n,%) | 47 (8.1) | 242 (37.2) | 0.740 | 47 (12.2) | 20 (13.0) | 0.023 |
| Chronic liver disease (n,%) | 22 (3.8) | 35 (5.4) | 0.076 | 18 (4.7) | 17 (4.4) | 0.012 |
| Chronic kidney disease (n,%) | 27 (4.7) | 37 (5.7) | 0.046 | 24 (6.2) | 24 (6.2) | 0.001 |
| Prostate hyperplasia (n,%) | 7 (1.2) | 20 (3.1) | 0.129 | 7 (1.8) | 6 (1.6) | 0.020 |
| Urolithiasis (n,%) | 9 (1.6) | 6 (1.4) | 0.014 | 6 (1.6) | 8 (2.1) | 0.039 |
| Vesicoureteral disease (n,%) | 19 (3.3) | 38 (5.8) | 0.123 | 16 (4.2) | 21 (5.5) | 0.061 |
| Catheterization (n,%) | 252 (43.4) | 318 (48.8) | 0.108 | 184 (47.8) | 183 (47.5) | 0.005 |
| Total time of indwelling catheter, ×hours (Mean, SD) | 1.41 (2.62) | 2.13 (4.03) | 0.211 | 1.73 (3.04) | 1.90 (3.47) | 0.050 |
| Tumor (n,%) | 44 (7.6) | 68 (10.4) | 0.100 | 39 (10.1) | 38 (9.9) | 0.009 |
| Operation |  |  |  |  |  |  |
| Fracture type |  |  |  |  |  |  |
| Femoral neck fracture (n,%) | 348 (60.0) | 290 (44.5) | 0.272 | 191 (49.6) | 190 (49.4) | 0.017 |
| Intertrochanteric fracture (n,%) | 200 (34.5) | 319 (49.0) |  | 165 (42.9) | 171 (44.4) |  |
| Subtrochanteric fracture (n,%) | 32 (5.5) | 42 (6.5) |  | 29 (7.5) | 24 (6.2) |  |
| Treatment Type |  |  |  |  |  |  |
| Total hip arthroplasty (n,%) | 88 (15.2) | 68 (10.4) | 0.138 | 51 (13.2) | 38 (9.9) | 0.051 |
| Hemiarthroplasty (n,%) | 133 (22.9) | 164 (25.2) |  | 101 (26.2) | 105 (27.3) |  |
| Intramedullary nail (n,%) | 158 (27.2) | 256 (39.3) |  | 132 (34.3) | 136 (35.3) |  |
| Plate/screw (n,%) | 63 (10.9) | 104 (16.0) |  | 50 (13.0) | 59 (15.3) |  |
| Multiple screws (n,%) | 138 (23.8) | 59 (9.1) |  | 51 (13.2) | 47 (12.2) |  |
| Bedridden time，×days (Mean, SD) | 5.38 (3.32) | 6.33 (4.45) | 0.142 | 5.76 (3.44) | 5.80 (4.20) | 0.009 |
| Surgery time, ×hours (Mean, SD) | 1.59 (0.74) | 1.72 (0.85) | 0.160 | 1.69 (0.83) | 1.70 (0.79) | 0.003 |
| ASA |  |  |  |  |  |  |
| Ⅲ-Ⅴ(n,%) | 291 (50.2) | 406 (62.4) | 0.247 | 237 (61.6) | 219 (56.9)) | 0.095 |
| Ⅰ-Ⅱ(n,%) | 289 (49.8) | 245(37.6) |  | 148 (38.4) | 166 (43.1) |  |

Abbreviations: ASA, American Society of Anesthesiologists score

**eTable4 Univariate and multivariate regression analyses of risk factors for UTIs (ALB-UTIs)**

| Characteristics | Univariate | | | Multivariate | | |
| --- | --- | --- | --- | --- | --- | --- |
|  | OR | 95%CI | P-value | OR | 95%CI | P-value |
| Age | 1.05 | 1.03-1.06 | <0.001 | 1.02 | 1.01-1.04 | 0.039 |
| Female gender | 2.21 | 1.65-2.97 | <0.001 | 2.75 | 1.85-4.08 | <0.001 |
| Smoking | 0.57 | 0.38-0.85 | 0.006 | 1.181 | 0.68-2.06 | 0.556 |
| Alcohol | 0.50 | 0.31-0.82 | 0.006 | 0.99 | 0.49-1.99 | 0.972 |
| Hypertension | 2.06 | 1.57-2.71 | <0.001 | 1.33 | 0.96-1.84 | 0.086 |
| Diabetes | 1.98 | 2.23-3.97 | <0.001 | 2.59 | 1.87-3.58 | <0.001 |
| Chronic liver disease | 1.56 | 0.88-2.77 | 0.13 | <NA> | <NA> | <NA> |
| Chronic kidney disease | 3.36 | 2.02-5.60 | <0.001 | 2.23 | 1.18-4.20 | 0.013 |
| Prostate hyperplasia | 2.72 | 1.26-5.87 | 0.01 | 4.44 | 1.71-11.57 | 0.002 |
| Urolithiasis | 2.69 | 1.05-6.88 | 0.04 | 2.26 | 0.70-7.58 | 0.168 |
| Vesicoureteral disease | 3.98 | 2.33-6.824 | <0.001 | 1.99 | 0.99-4.01 | 0.054 |
| Catheterization | 2.28 | 1.74-3.00 | <0.001 | 1.12 | 0.76-1.62 | 0.572 |
| Total time of indwelling catheter | 1.21 | 1.16-1.26 | <0.001 | 1.15 | 1.09-1.22 | <0.001 |
| Tumor | 0.87 | 0.52-1.36 | 0.479 | <NA> | <NA> | <NA> |
| Fracture type | 0.627 | 0.507-0.775 | <0.001 | 0.55 | 0.41-0.74 | <0.001 |
| Treatment Type | 0.88 | 0.79-0.98 | 0.02 | 0.87 | 0.74-1.02 | 0.092 |
| Bedridden time | 1.08 | 1.05-1.12 | <0.001 | 1.05 | 1.01-1.09 | 0.010 |
| Surgery time | 1.17 | 1.00-1.36 | 0.054 | <NA> | <NA> | <NA> |
| ASA | 1.46 | 1.11-1.92 | 0.006 | 0.72 | 0.52-1.01 | 0.056 |
| ALB(< 35 g/L) | 2.00 | 1.30-3.08 | <0.001 | 1.59 | 1.11-2.27 | 0.001 |

Abbreviations: ASA, American Society of Anesthesiologists score; ALB, Albumin

**eTable5 Patient characteristics before and after propensity score matching by ALB clinical cutoffs (ALB-UTIs)**

| Characteristics | Before matching | | | After matching | | |
| --- | --- | --- | --- | --- | --- | --- |
|  | ALB＜35  (n= 257) | ALB ≥ 35  (n= 974) | SMD | ALB＜35  (n= 239) | ALB ≥ 35  (n= 239) | SMD |
| Demographic |  |  |  |  |  |  |
| Age, × years (Mean, SD) | 79.60(9.56) | 73.57(9.21) | <0.001 | 78.89(9.30) | 78.56(9.17) | 0.036 |
| Female gender (n,%) | 147 (57.2) | 598 (61.4) | 0.221 | 144 (60.3) | 137 (57.3) | 0.059 |
| Smoking (n,%) | 31 (12.1) | 178 (18.3) | 0.018 | 30 (12.6) | 26 (10.9) | 0.052 |
| Alcohol (n,%) | 22 (8.6) | 121 (12.4) | 0.086 | 21 (8.8) | 20 (8.4) | 0.015 |
| Comorbidities |  |  |  |  |  |  |
| Hypertension (n,%) | 139 (54.1) | 486 (49.9) | 0.233 | 132 (55.2) | 131 (54.8) | 0.008 |
| Diabetes (n,%) | 52 (20.2) | 237 (24.3) | 0.168 | 51 (21.3) | 53 (22.2) | 0.020 |
| Chronic liver disease (n,%) | 17 (6.6) | 40 (4.1) | 0.089 | 15 (6.3) | 20 (8.4) | 0.080 |
| Chronic kidney disease (n,%) | 16 (6.2) | 48 (4.9) | 0.405 | 15 (6.3) | 13 (5.4) | 0.036 |
| Prostate hyperplasia (n,%) | 9 (3.5) | 18 (1.8) | 0.108 | 7(2.9) | 8 (3.3) | 0.024 |
| Urolithiasis (n,%) | 3 (1.2) | 15 (1.5) | 0.658 | 3 (1.3) | 5 (2.1) | 0.065 |
| Vesicoureteral disease (n,%) | 15 (5.8) | 42 (4.3) | 0.301 | 14 (5.90) | 17 (7.10) | 0.051 |
| Catheterization (n,%) | 124 (48.2) | 446 (45.8) | 0.481 | 113 (47.3) | 102 (42.7) | 0.092 |
| Total time of indwelling catheter, ×hours (Mean, SD) | 2.60 (4.99) | 1.58 (2.88) | <0.001 | 2.15 (3.71) | 2.28 (4.40) | 0.034 |
| Tumor (n,%) | 21 (8.2) | 91 (9.3) | 0.562 | 20 (8.4) | 19 (7.9) | 0.015 |
| Operation |  |  |  |  |  |  |
| Fracture type |  |  |  |  |  |  |
| Femoral neck fracture (n,%) | 90 (35.0) | 548 (56.3) | <0.001 | 88 (36.8) | 96 (40.20) | 0.085 |
| Intertrochanteric fracture (n,%) | 148 (57.6) | 371 (38.1) |  | 133 (55.6) | 129 (54.0) |  |
| Subtrochanteric fracture (n,%) | 19 (7.4) | 55 (5.6) |  | 18(7.5) | 14 (5.9) |  |
| Treatment Type |  |  |  |  |  |  |
| Total hip arthroplasty (n,%) | 23 (8.9) | 133 (13.7) | 0.956 | 23 (9.6) | 24 (10.0) | 0.071 |
| Hemiarthroplasty (n,%) | 52 (20.2) | 245 (25.2) |  | 50 (20.9) | 59 (24.7) |  |
| Intramedullary nail (n,%) | 114 (44.4) | 300 (30.8) |  | 102 (42.7) | 101 (42.3) |  |
| Plate/screw (n,%) | 49 (19.1) | 118 (12.1) |  | 45 (18.8) | 34 (14.2) |  |
| Multiple screws (n,%) | 199 (7.4) | 178 (18.3) |  | 19 (7.9) | 21 (8.8) |  |
| Bedridden time，×days (Mean, SD) | 6.74 (5.04) | 5.65(3.62) | <0.001 | 6.48 (3.94) | 6.15 (4.24) | 0.080 |
| Surgery time, ×hours (Mean, SD) | 1.75 (0.85) | 1.63 (0.78) | 0.032 | 1.75 (0.86) | 1.69 (0.80) | 0.072 |
| ASA |  |  |  |  |  |  |
| Ⅲ-Ⅴ(n,%) | 181 (70.4) | 516 (53.0) | <0.001 | 165 (69.0) | 170 (71.1) | 0.046 |
| Ⅰ-Ⅱ(n,%) | 76 (29.6) | 458 (47.0) |  | 74 (31.0) | 69 (28.9) |  |

Abbreviations: ASA, American Society of Anesthesiologists score; ALB, Albumin

**eTable6 Univariate and multivariate regression analyses of risk factors for UTIs (GLB-UTIs)**

| Characteristics | Univariate | | | Multivariate | | |
| --- | --- | --- | --- | --- | --- | --- |
|  | OR | 95%CI | P-value | OR | 95%CI | P-value |
| Age | 1.05 | 1.03-1.06 | <0.001 | 1.03 | 1.01-1.05 | 0.003 |
| Female gender | 2.21 | 1.65-2.97 | <0.001 | 2.50 | 1.68-3.72 | <0.001 |
| Smoking | 0.57 | 0.38-0.85 | 0.006 | 1.10 | 0.63-1.92 | 0.735 |
| Alcohol | 0.50 | 0.31-0.82 | 0.006 | 1.00 | 0.50-2.03 | 0.997 |
| Hypertension | 2.06 | 1.57-2.71 | <0.001 | 1.24 | 0.89-1.71 | 0.203 |
| Diabetes | 1.98 | 2.23-3.97 | <0.001 | 2.45 | 1.77-3.40 | <0.001 |
| Chronic liver disease | 1.56 | 0.88-2.77 | 0.13 | <NA> | <NA> | <NA> |
| Chronic kidney disease | 3.36 | 2.02-5.60 | <0.001 | 2.13 | 1.13-4.02 | 0.019 |
| Prostate hyperplasia | 2.72 | 1.26-5.87 | 0.01 | 4.82 | 1.87-12.40 | 0.001 |
| Urolithiasis | 2.69 | 1.05-6.88 | 0.04 | 2.56 | 0.78-8.47 | 0.122 |
| Vesicoureteral disease | 3.98 | 2.33-6.824 | <0.001 | 2.04 | 1.01-4.14 | 0.048 |
| Catheterization | 2.28 | 1.74-3.00 | <0.001 | 1.09 | 0.74-1.61 | 0.649 |
| Total time of indwelling catheter | 1.21 | 1.16-1.26 | <0.001 | 1.17 | 1.10-1.24 | <0.001 |
| Tumor | 0.87 | 0.52-1.36 | 0.479 | <NA> | <NA> | <NA> |
| Fracture type | 0.627 | 0.507-0.775 | <0.001 | 0.48 | 0.36-0.65 | <0.001 |
| Treatment Type | 0.88 | 0.79-0.98 | 0.02 | 0.86 | 0.73-1.02 | 0.088 |
| Bedridden time | 1.08 | 1.05-1.12 | <0.001 | 1.06 | 1.02-1.10 | 0.003 |
| Surgery time | 1.17 | 1.00-1.36 | 0.054 | <NA> | <NA> | <NA> |
| ASA | 1.46 | 1.11-1.92 | 0.006 | 0.757 | 0.54-1.06 | 0.101 |
| GLB(> 30 g/L) | 1.65 | 1.19-2.29 | 0.003 | 1.35 | 2.62-6.18 | <0.001 |

Abbreviations: ASA, American Society of Anesthesiologists score; GLB, Globulin

**eTable7 Patient characteristics before and after propensity score matching by GLB clinical cutoffs (GLB-UTIs)**

| Characteristics | Before matching | | | After matching | | |
| --- | --- | --- | --- | --- | --- | --- |
|  | GLB ≤ 30  (n=911) | GLB > 30  (n=320) | SMD | GLB ≤ 30  (n=314) | GLB > 30  (n=314) | SMD |
| Demographic |  |  |  |  |  |  |
| Age, × years (Mean, SD) | 74.48 (9.65) | 75.82 (9.39) | 0.140 | 76.07 (9.24) | 75.69 (9.42) | 0.040 |
| Female gender (n,%) | 532 (58.4) | 213 (66.6) | 0.169 | 220 (70.1) | 208 (66.2) | 0.082 |
| Smoking (n,%) | 159 (17.5) | 50 (15.6) | 0.049 | 47 (15.0) | 49 (15.6) | 0.018 |
| Alcohol (n,%) | 119 (13.1) | 24 (7.5) | 0.184 | 22 (7.0) | 27 (7.6) | 0.024 |
| Comorbidities |  |  |  |  |  |  |
| Hypertension (n,%) | 431 (47.3) | 194 (60.6) | 0.269 | 189 (60.2) | 188 (69.9) | 0.006 |
| Diabetes (n,%) | 200 (22.0) | 89 (27.8) | 0.136 | 88 (28.0) | 87 (27.7) | 0.007 |
| Chronic liver disease (n,%) | 39 (4.3) | 18 (5.6) | 0.062 | 14 (4.5) | 17 (5.4) | 0.044 |
| Chronic kidney disease (n,%) | 38 (4.2) | 26 (9.1) | 0.165 | 23 (7.3) | 22 (7.0) | 0.012 |
| Prostate hyperplasia (n,%) | 22 (2.4) | 5 (1.6) | 0.061 | 4 (1.3) | 5 (1.6) | 0.027 |
| Urolithiasis (n,%) | 9 (1.0) | 9 (2.8) | 0.134 | 5 (1.6) | 7 (2.2) | 0.046 |
| Vesicoureteral disease (n,%) | 40 (4.4) | 17 (5.3) | 0.043 | 16 (5.1) | 15 (4.8) | 0.015 |
| Catheterization (n,%) | 415 (45.6) | 155 (48.4) | 0.058 | 152 (48.4) | 151 (48.1) | 0.006 |
| Total time of indwelling catheter, ×hours (Mean, SD) | 1.73 (3.19) | 1.96 (4.12) | 0.061 | 2.11 (3.92) | 1.93 (4.13) | 0.044 |
| Tumor (n,%) | 80 (8.8) | 32(10.0) | 0.042 | 29 (9.2) | 32 (10.2) | 0.032 |
| Operation |  |  |  |  |  |  |
| Fracture type |  |  |  |  |  |  |
| Femoral neck fracture (n,%) | 459 (50.4) | 179 (55.9) | 0.086 | 158 (50.3) | 175 (55.7) | 0.098 |
| Intertrochanteric fracture (n,%) | 398 (43.7) | 121 (37.8) |  | 134 (42.7) | 119 (55.7) |  |
| Subtrochanteric fracture (n,%) | 54 (5.9) | 20 (6.3) |  | 122 (7.0) | 20 (6.4) |  |
| Treatment Type |  |  |  |  |  |  |
| Total hip arthroplasty (n,%) | 110 (12.1) | 46 (14.4) | 0.167 | 40 (12.7) | 45 (14.3) | 0.042 |
| Hemiarthroplasty (n,%) | 204 (22.4) | 93 (29.1) |  | 81 (25.8) | 90 (28.7) |  |
| Intramedullary nail (n,%) | 314 (34.5) | 100 (31.3) |  | 112 (35.7) | 98 (31.2) |  |
| Plate/screw (n,%) | 129 (14.2) | 38 (11.9) |  | 41 (13.1) | 38 (12.1) |  |
| Multiple screws (n,%) | 154 (16.9) | 43 (13.4) |  | 40 (12.7) | 43 (13.7) |  |
| Bedridden time，×days (Mean, SD) | 5.83 (4.20) | 6.01 (3.29) | 0.048 | 6.03 (4.08) | 5.98 (3.29) | 0.014 |
| Surgery time, ×hours (Mean, SD) | 1.65 (0.80) | 1.67 (0.80) | 0.019 | 1.75 (0.95) | 1.65 (0.77) | 0.115 |
| ASA |  |  |  |  |  |  |
| Ⅲ-Ⅴ(n,%) | 494 (54.2) | 203 (63.4) | 0.188 | 191 (90.8) | 197 (62.7) | 0.039 |
| Ⅰ-Ⅱ(n,%) | 417 (45.8) | 117 (36.6) |  | 123 (39.2) | 117 (37.3) |  |

Abbreviations: ASA, American Society of Anesthesiologists score; GLB, Globulin

**eTable8 Univariate and multivariate regression analyses of risk factors for UTIs (HDL-UTIs)**

| Characteristics | Univariate | | | Multivariate | | |
| --- | --- | --- | --- | --- | --- | --- |
|  | OR | 95%CI | P-value | OR | 95%CI | P-value |
| Age | 1.05 | 1.03-1.06 | <0.001 | 1.03 | 1.01-1.05 | 0.001 |
| Female gender | 2.21 | 1.65-2.97 | <0.001 | 2.79 | 1.87-4.12 | <0.001 |
| Smoking | 0.57 | 0.38-0.85 | 0.006 | 1.18 | 0.68-2.06 | 0.550 |
| Alcohol | 0.50 | 0.31-0.82 | 0.006 | 0.92 | 0.46-1.85 | 0.815 |
| Hypertension | 2.06 | 1.57-2.71 | <0.001 | 1.30 | 0.94-1.79 | 0.115 |
| Diabetes | 1.98 | 2.23-3.97 | <0.001 | 2.43 | 1.76-3.37 | <0.001 |
| Chronic liver disease | 1.56 | 0.88-2.77 | 0.13 | <NA> | <NA> | <NA> |
| Chronic kidney disease | 3.36 | 2.02-5.60 | <0.001 | 2.29 | 1.21-4.32 | 0.011 |
| Prostate hyperplasia | 2.72 | 1.26-5.87 | 0.01 | 4.50 | 1.76-11.53 | 0.002 |
| Urolithiasis | 2.69 | 1.05-6.88 | 0.04 | 2.58 | 0.78-8.47 | 0.119 |
| Vesicoureteral disease | 3.98 | 2.33-6.824 | <0.001 | 1.97 | 0.98-3.95 | 0.057 |
| Catheterization | 2.28 | 1.74-3.00 | <0.001 | 1.11 | 0.76-1.62 | 0.606 |
| Total time of indwelling catheter | 1.21 | 1.16-1.26 | <0.001 | 1.16 | 1.09-1.23 | <0.001 |
| Tumor | 0.87 | 0.52-1.36 | 0.479 | <NA> | <NA> | <NA> |
| Fracture type | 0.627 | 0.507-0.775 | <0.001 | 0.53 | 0.40-0.71 | <0.001 |
| Treatment Type | 0.88 | 0.79-0.98 | 0.02 | 0.87 | 0.74-1.03 | 0.1.1 |
| Bedridden time | 1.08 | 1.05-1.12 | <0.001 | 1.06 | 1.02-1.10 | 0.002 |
| Surgery time | 1.17 | 1.00-1.36 | 0.054 | <NA> | <NA> | <NA> |
| ASA | 1.46 | 1.11-1.92 | 0.006 | 0.74 | 0.53-1.04 | 0.079 |
| HDL(< 1.00 mmol/L) | 1.47 | 1.10-1.96 | 0.009 | 1.19 | 0.85-1.67 | 0.300 |

Abbreviations: ASA, American Society of Anesthesiologists score; HDL, High-density lipoprotein

**eTable9 Patient characteristics before and after propensity score matching by HDL clinical cutoffs (HDL-UTIs)**

| Characteristics | Before matching | | | After matching | | |
| --- | --- | --- | --- | --- | --- | --- |
|  | HDL < 1.00  (n=322) | HDL ≥ 1.00  (n=909) | SMD | HDL < 1.00  (n=318) | HDL ≥ 1.00  (n=318) | SMD |
| Demographic |  |  |  |  |  |  |
| Age, × years (Mean, SD) | 75.38 (9.66) | 74.64 (9.57) | 0.077 | 75.36 (9.67) | 75.04 (9.72) | 0.033 |
| Female gender (n,%) | 175 (54.3) | 570 (62.7) | 0.170 | 220 (70.1) | 208 (66.2) | 0.044 |
| Smoking (n,%) | 59 (18.3) | 150 (16.5) | 0.048 | 47 (15.0) | 49 (15.6) | 0.024 |
| Alcohol (n,%) | 40 (12.4) | 103 (11.3) | 0.034 | 22 (7.0) | 27 (7.6) | 0.028 |
| Comorbidities |  |  |  |  |  |  |
| Hypertension (n,%) | 178 (55.3) | 447 (49.2) | 0.122 | 189 (60.2) | 188 (69.9) | 0.025 |
| Diabetes (n,%) | 89 (27.6) | 200 (22.0) | 0.131 | 88 (28.0) | 87 (27.7) | 0.043 |
| Chronic liver disease (n,%) | 24 (7.5) | 33 (3.6) | 0.167 | 14 (4.5) | 17 (5.4) | 0.012 |
| Chronic kidney disease (n,%) | 25 (7.8) | 39 (4.3) | 0.146 | 23 (7.3) | 22 (7.0) | 0.048 |
| Prostate hyperplasia (n,%) | 7 (2.2) | 20 (2.2) | 0.002 | 4 (1.3) | 5 (1.6) | 0.022 |
| Urolithiasis (n,%) | 7 (2.2) | 11 (1.2) | 0.075 | 5 (1.6) | 7 (2.2) | 0.043 |
| Vesicoureteral disease (n,%) | 21 (6.5) | 36 (4.0) | 0.115 | 16 (5.1) | 15 (4.8) | 0.013 |
| Catheterization (n,%) | 153 (47.5) | 417 (45.9) | 0.033 | 152 (48.4) | 151 (48.1) | 0.006 |
| Total time of indwelling catheter, ×hours (Mean, SD) | 2.21 (3.61) | 1.64 (3.39) | 0.164 | 2.12 (3.49) | 1.90 (3.61) | 0.064 |
| Tumor (n,%) | 24 (7.5) | 88 (9.7) | 0.080 | 29 (9.2) | 32 (10.2) | 0.035 |
| Operation |  |  |  |  |  |  |
| Fracture type |  |  |  |  |  |  |
| Femoral neck fracture (n,%) | 129 (40.1) | 509 (56.0) | 0.270 | 158 (50.3) | 175 (55.7) | 0.016 |
| Intertrochanteric fracture (n,%) | 173 (53.7) | 346 (38.1) |  | 134 (42.7) | 119 (55.7) |  |
| Subtrochanteric fracture (n,%) | 20 (6.2) | 54 (5.9) |  | 122(7.0) | 20 (6.4) |  |
| Treatment Type |  |  |  |  |  |  |
| Total hip arthroplasty (n,%) | 36 (11.2) | 120 (13.2) | 0.016 | 40 (12.7) | 45 (14.3) | 0.008 |
| Hemiarthroplasty (n,%) | 60 (18.6) | 237 (26.1) |  | 81 (25.8) | 90 (28.7) |  |
| Intramedullary nail (n,%) | 140 (43.5) | 274 (30.1) |  | 112 (35.7) | 98 (31.2) |  |
| Plate/screw (n,%) | 48 (14.9) | 119 (13.1) |  | 41 (13.1) | 38 (12.1) |  |
| Multiple screws (n,%) | 38 (11.8) | 159 (17.5) |  | 40 (12.7) | 43 (13.7) |  |
| Bedridden time，×days (Mean, SD) | 5.92 (4.29) | 5.87 (3.87) | 0.012 | 5.86 (4.17) | 5.96 (3.55) | 0.027 |
| Surgery time, ×hours (Mean, SD) | 1.72 (0.96) | 1.63 (0.73) | 0.095 | 1.71 (0.95) | 1.69 (0.77) | 0.026 |
| ASA |  |  |  |  |  |  |
| Ⅲ-Ⅴ(n,%) | 183 (56.8) | 514 (56.5) | 0.006 | 191 (90.8) | 197(62.7) | 0.013 |
| Ⅰ-Ⅱ(n,%) | 139 (43.2) | 395 (43.5) |  | 123(39.2) | 117(37.3) |  |

Abbreviations: ASA, American Society of Anesthesiologists score; HDL, High-density lipoprotein
